# Identification of a new anoikis-related gene signature for prognostic significance in head and neck squamous carcinomas

**DOI:** 10.1097/MD.0000000000034790

**Published:** 2023-09-08

**Authors:** Zhengyu Wei, Chongchang Zhou, Yi Shen, Hongxia Deng, Zhisen Shen

**Affiliations:** a Department of Otorhinolaryngology Head and Neck Surgery, The Affiliated Lihuili Hospital, Ningbo University, Ningbo, China; b Department of Otorhinolaryngology Head and Neck Surgery, Ningbo Medical Centre Lihuili Hospital, Ningbo, China; c Health Science Center, Ningbo University, Ningbo, China.

**Keywords:** anoikis, chemotherapy, head and neck squamous cell carcinoma, immunotherapy, prognosis

## Abstract

Anoikis, a mode of programmed cell death, is essential for normal development and homeostasis in the organism and plays an important role in the onset and progression of cancers. The authors of this research sought to establish a gene signature associated with anoikis to predict therapy outcomes and patient prognosis for individuals with head and neck squamous cell carcinoma (HNSCC). Transcriptome data of anoikis-related genes (ARGs) in individuals with HNSCC were retrieved from public databases to aid in the formulation of the gene signature. A novel ARG signature was then created using a combination of the Least Absolute Shrinkage and Selection Operator regression and Cox regression analysis. The relationship between ARGs and tumor immune microenvironment in HNSCC was explored using single-cell analysis. HNSCC individuals were classified into high-risk and low-risk groups as per the median value of risk score. The study also investigated the variations in the infiltration status of immune cells, tumor microenvironment, sensitivity to immunotherapy and chemotherapeutics, as well as functional enrichment between the low-risk and high-risk categories. A total of 18 ARGs were incorporated in the formulation of the signature. Our signature’s validity as a standalone predictive predictor was validated by multivariate Cox regression analysis and Kaplan–Meier survival analysis. Generally, the prognosis was worse for high-risk individuals. Subjects in the low-risk groups had a better prognosis and responded in a better way to combination immunotherapy, had higher immunological ratings and activity levels, and had more immune cell infiltration. In addition, gene set enrichment analysis findings showed that the low-risk subjects exhibited heightened activity in several immune-related pathways. However, the high-risk patients responded better to chemotherapy. The aim of this research was to develop a new ARG signature to predict the prognosis and sensitivity to immunotherapeutic and chemotherapeutic schemes for HNSCC patient. As a result, this could help spur the creation of new chemotherapeutics and immunotherapeutic approaches for patients with HNSCC.

## 1. Introduction

Head and neck squamous cell carcinoma (HNSCC) ranks sixth in prevalence among all cancers,^[1]^ with more than 600,000 new cases of HNSCC occurring and over 300,000 deaths every year.^[2,3]^ Several etiological factors for HNSCC are known, including drinking, smoking, and human papillomavirus infection.^[4]^ When compared to HNSCC caused by smoking and alcohol consumption, HNSCC caused by human papillomavirus has a superior clinical prognosis and responds better to chemoradiation.^[5]^ Patients with HNSCC whose cancer has spread to the cervical lymph nodes have a 50% lower 5-year mortality rate than those whose cancer has not spread to the cervical lymph nodes.^[6,7]^ Surgery, radiotherapy, chemotherapy, and biotherapy are the primary therapeutic strategies for HNSCC.^[8]^ Despite immunotherapy having achieved rapid clinical successes in treating HNSCC,^[9]^ the survival rate in patients with HNSCC remains unsatisfactory, and the 5-year survival rates for HNSCC patients are approximately 40% to 50%.^[10,11]^ Given this, novel therapeutic strategies are urgently needed to improve HNSCC survival rates.

Integrins play a major part in the transmission of signals within cells, attachment to extracellular matrix (ECM), and cell-to-cell interactions.^[12]^ Apoptosis caused by a loss of integrin-mediated attachment to the ECM is known as anoikis.^[13]^ The process of anoikis is essential for maintaining normal cell and tissue homeostasis, maintaining a dynamic balance between proliferation, differentiation, and apoptosis.^[14]^ Tumor cells, in contrast to healthy cells, can evolve a number of coping mechanisms to avoid anoikis, such as delaying anoikis through autophagy and reciprocal phagocytosis and increasing the survival rate of metastatic cells, both of which add to tumor development and progression.^[15,16]^ HNSCC is among the tumors whose cells are increasingly characterized by their resistance to anoikis.^[17]^ Anoikis resistance in HNSCC was found to be associated with the VEGFA-STAT3-KLF4-CDKN1A signaling pathway.^[18]^ Anoikis resistance in HNSCC has also been linked to fibronectin.^[19]^ The cause of anoikis resistance in HNSCC has been the subject of numerous research.^[18]^ The process of epithelium cells changing into mesenchymal cells in response to certain normal and pathogenic stimuli is a frequent event in the development of HNSCC and is referred to as epithelial-mesenchymal transition. Many researchers now believe that the epithelial-mesenchymal transition process is a significant contributor to cancer cells developing anoikis resilience.^[18]^ It is still unknown, however, how anoikis-related genes (ARGs) factor into HNSCC prognosis projection.

In this research, Least Absolute Shrinkage and Selection Operator (LASSO) regression analysis was utilized in the development of an ARG signature. We conducted multiple analyses, including decision curve analysis, Kaplan–Meier analysis, C-index, time-dependent receiver operating characteristic (ROC) curve analysis, as well as univariate and multivariate Cox regression analyses to examine ARG signature’s predictive significance for HNSCC patients. Additionally, we investigated whether or not there was a link between the ARGs signature and infiltration of immune cells, the TME (tumor microenvironment), and the curative efficacy of immunotherapy and chemotherapeutics. Functional enrichment analysis was performed in order to learn more about the molecular role played by the ARGs pattern in the HNSCC.

## 2. Materials and Methods

### 2.1. HNSCC datasets

**Figure 1.**
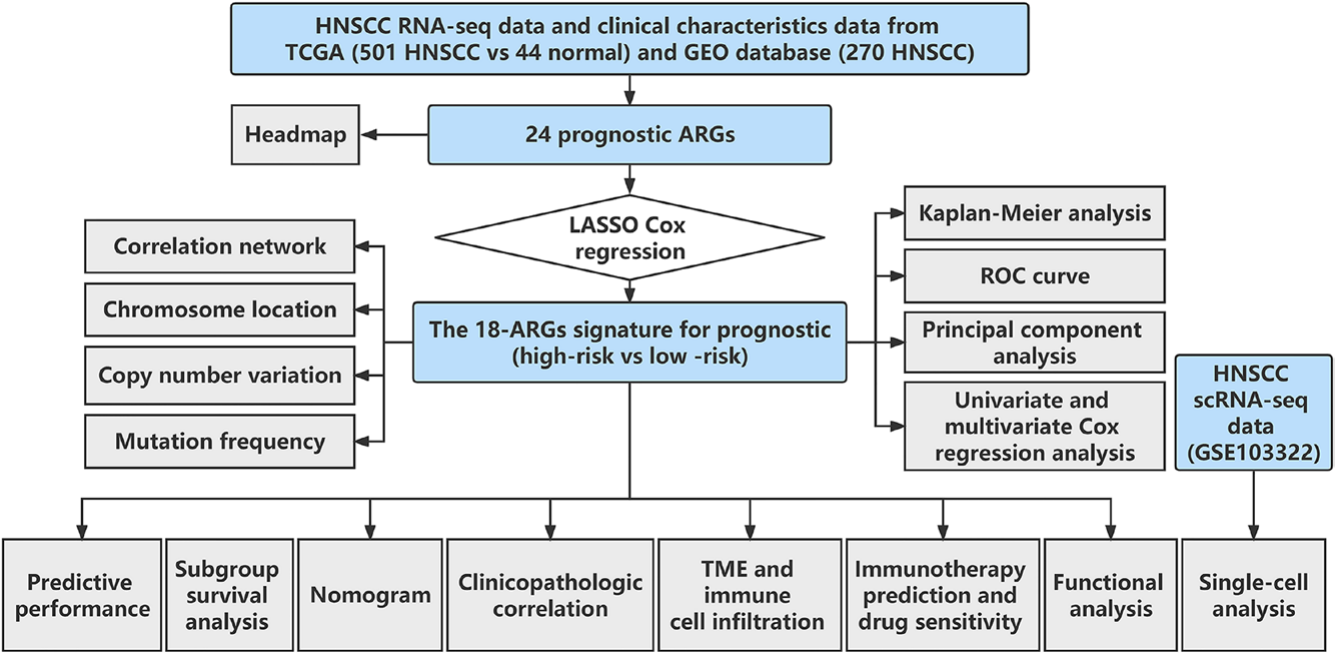
The study’s flow chart. ARGs = anoikis-related genes, GEO = gene expression omnibus, HNSCC = head and neck squamous carcinoma, ROC = receiver operating characteristic, TCGA = the cancer genome atlas, TME = tumor microenvironment.

Figure 1 displays the study’s flowchart. The training cohort consisted of 501 HNSCC cases and 44 healthy tissues acquired from The Cancer Genome Atlas (TCGA). The validation cohort GSE65858 was downloaded from the Gene Expression Omnibus (GEO) collection which consisted of 270 HNSCC cases. Sex, histopathological grade, age, metastasis status, T stage, survival status, clinical stage, and N stage were among the clinical data that were retrieved (Table 1). A publicly available single-cell RNA sequencing dataset (GSE103322) was also used to investigate the relationship between ARGs and tumor immune microenvironment (TME) in HNSCC.^[20]^ The human ethics committees of the Ningbo Medical Centre Lihuili Hospital approved these experimental protocols (number: KY2023SL016).

**Table 1:** Clinical characteristics of the patients with head and neck squamous carcinomas in this study.

Covariates	Type	TCGA cohort		GSE65858 cohort	
		Number	Percent	Number	Percent
Age	≤60	246	49.10	130	48.15
	>60	255	50.90	140	51.85
Gender	Female	133	26.55	47	17.41
	Male	368	73.45	223	82.59
Histologic grade	G1–2	359	71.66	–	–
	G3–4	121	24.15	–	–
	Unknown	19	3.79	–	–
T stage	T1–2	177	35.33	115	42.59
	T3–4	309	61.68	155	57.41
	Unknown	15	2.99	–	–
N stage	N0	239	47.70	94	34.81
	N1–3	240	47.90	176	65.19
	Unknown	22	4.39	–	–
Metastasis status	Yes	25	4.99	–	–
	No	471	94.01	–	–
	Unknown	5	1.00	–	–
Clinical stage	Stage I–II	114	22.75	55	20.37
	Stage III–IV	373	74.45	215	79.63
	Unknown	14	2.79	–	–
Survival status	Dead	217	43.31	94	34.81
	Alive	284	56.69	176	65.19

TCGA = The Cancer Genome Atlas.

### 2.2. Development of the ARG signature for HNSCC subjects

A total of 337 ARGs with a relevance score of more than 1 were culled from the MSigDB database. Differences in ARG expression were examined between normal and HNSCC cells using the “limma” R program. Identification of the predictive ARGs was done via univariate Cox regression analysis using the “survival” R program at *P* values less than .05. Next, to determine the optimal prognostic ARGs in constructing the prognostic ARG signature, we utilized the “glmnet” R package to execute the LASSO regression approach with 10-fold cross-validation. Expression levels of ARGs and corresponding coefficients were used to determine risk scores for HNSCC cases. The formula is shown below:


$$\begin{array}{l} Risk\;score=\overset{n}{\underset{i\;=\;1}{\sum }}coef\;\;f\;\;icient\times ARG\;expression \end{array}$$

### 2.3. The landscape of ARGs expression, copy number variation (CNV), and mutation analysis in HNSCC patients

Herein, we employed the “limma” R tool to measure the levels of expression of 18 ARGs included in the signature. The correlation network between the ARGs was established using Pearson correlation. CNV and mutation frequency analyses of ARGs were performed by “maftools” and “RCircos” R packages.

### 2.4. Single-cell analysis

The Tumor Immune Single-Cell Hub (TISCH, http://tisch.comp-genomics.org/) provided comprehensive annotations of cell types and enabled interactive visualization of single-cell transcriptomes. The relationship between ARGs and TME in HNSCC was explored using TISCH.

### 2.5. The prognostic prediction value of ARG signature

The median value of risk score was utilized in the classification of HNSCC individuals into high-risk and low-risk categories. Analysis of progression-free survival, disease-specific survival, and overall survival (OS) in HNSCC individuals was performed using Kaplan–Meier survival curves to determine whether or not a risk score was associated with these outcomes. Age, gender, histopathological grade, and clinical stage all played a role in the subset mortality study that was conducted. The signature’s prognosis forecast ability was determined by plotting ROC curves against time and calculating the area under the curve (AUC) values. A principal components analysis was carried out to assess the ARG signature’s ability to differentiate between groups. For this study, we analyzed the ARG signature’s predictive significance in patients with HNSCC using multivariate and univariate Cox regression analyses. Predictive precision was determined using concordance index calculations. The “ggDCA” R software was used to analyze the decision curve. To determine whether our ARG signature had a superior predictive ability for HNSCC patients, 5 published prognostic signatures, Jiang signature,^[21]^ Ming signature,^[22]^ Yang signature,^[23]^ Zhang signature,^[24]^ and Zhao signature,^[25]^ were compared with our signature according to parameters including time-dependent ROC, C-index, and restricted mean survival time (RMST). A nomogram was developed to predict the rate of OS based on grade, risk score, age, gender, and stage. We assessed the nomogram’s ability to predict outcomes using ROC and calibration curves.

### 2.6. Immune cell infiltration and TME

The estimate scores, stromal scores, tumor purity, and immunological scores of HNSCC individuals were determined using the ESTIMATE^[26]^ algorithms in R. In addition, single-sample gene set enrichment analysis (GSEA) was performed to compare the immune functions between the 2 groups. Twenty-two different immune cells’ infiltration abundance was studied using the CIBERSORT algorithm.^[27]^

### 2.7. Immunotherapy prediction and drug sensitivity analysis

Immunophenoscores (IPS) and Tumor Immune Dysfunction and Exclusion (TIDE, http://tide.dfci.harvard.edu/) scores were used to predict the immunotherapy response of HNSCC patients. The higher IPS predicted better immunotherapy response, whereas the higher TIDE scores indicated worse immunotherapy response. The half maximal inhibitory concentration (IC50) values were calculated for assessing the sensitivity of HNSCC patients to the chemotherapeutic drugs. For a given medication, a greater IC50 value indicated a lower sensitivity to that drug.

### 2.8. Functional enrichment analysis

By applying the “limma” R tool, which requires FDR *q* < 0.05 and |log2FC|≥1 to find differentially expressed genes (DEGs) between the 2 groups. Gene ontology and Kyoto Encyclopedia of Genes and Genomes pathway enrichment studies were performed according to DEGs. We used GSEA software (version 4.1.0) to analyze the data to distinguish between the pathways that were enriched in the low-risk and high-risk groups.^[28]^

### 2.9. Statistical analysis

R, version 4.1.0, was utilized for statistical analyses. The chi-square test was utilized to examine the similarity or dissimilarity of the groups with respect to the categorical variables. The correlation coefficient was determined using Pearson’s correlation coefficient. Survivability was evaluated with tools like the log-rank test and the Kaplan–Meier curve. The predictive value of variables was analyzed using univariate and multivariate Cox regression models. A *P* value less than .05 indicated statistical significance.

## 3. Results

### 3.1. Recognition of differentially expressed prognostic ARGs in HNSCC

**Figure 2.**
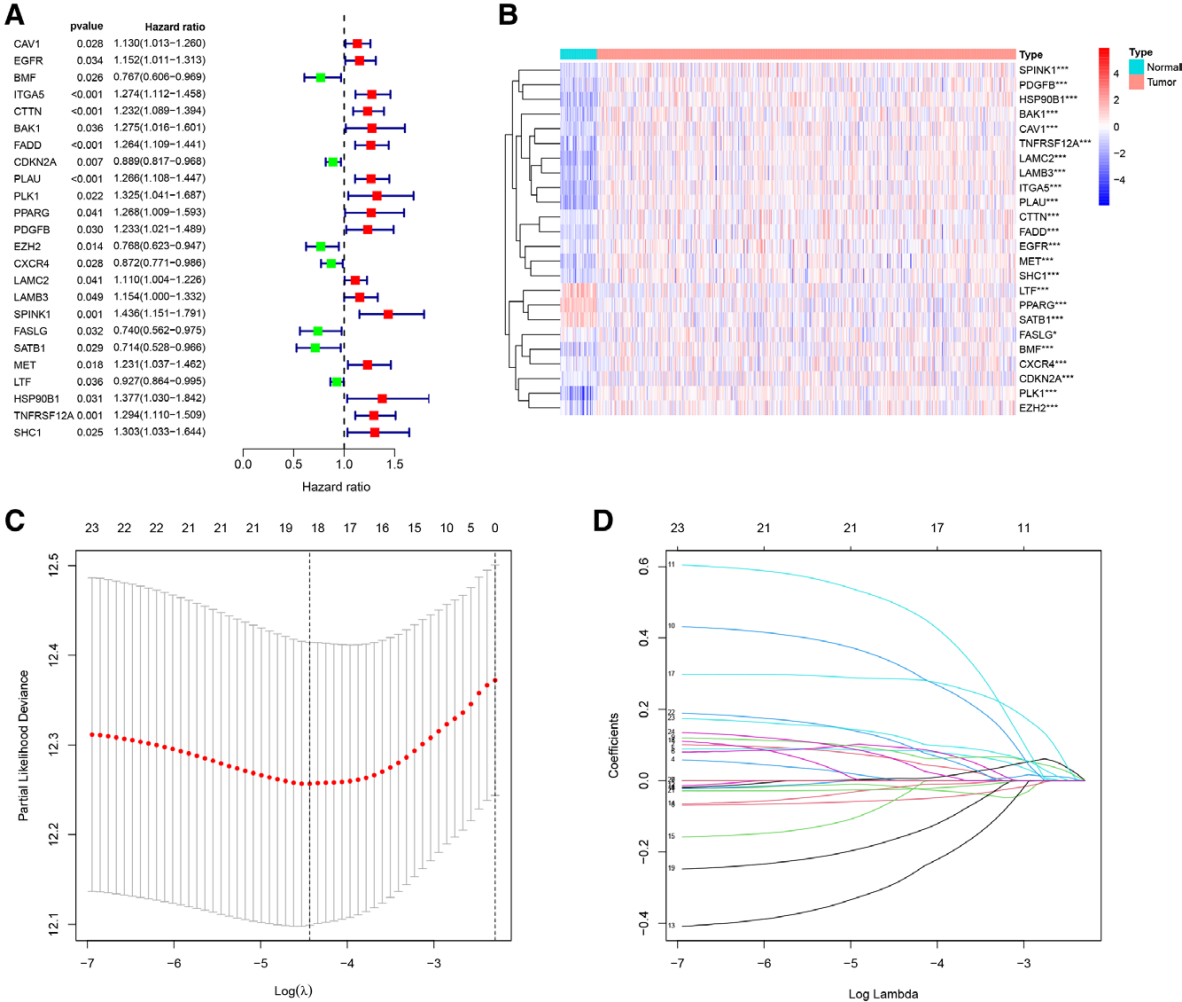
The development of the anoikis-related signature for patients with HNSCC. (A) The prognostic ARGs via univariate Cox regression analysis (*P* < .05). (B) The 24 ARGs differentially expressed between HNSCC and nearby healthy tissues, shown as a heatmap (red color depicts high expression, blue color depicts low expression). ***P < .001; **P < .01; *P < .05. (C) The optimal variable (λ) for the LASSO Cox regression model was chosen using minimal criterion. (D) The LASSO coefficient of the ARGs in the signature. ARGs = anoikis-related genes, HNSCC = head and neck squamous carcinoma, LASSO = Least Absolute Shrinkage and Selection Operator.

We conducted the comparison of the expression of 337 ARGs in 501 HNSCC samples to that of 44 control samples using data retrieved from the TCGA database. There were 106 ARGs that showed significant changes in expression levels in HNSCC. 24 predictive ARGs were then screened out using univariate Cox regression analysis, as illustrated in Figure 2A. We compared the transcript levels of 24 prognostic ARGs in 501 samples of HNSCC patients and 44 nearby healthy tissues in the heatmap (Fig. 2B).

### 3.2. Development of a prognostic ARG signature for HNSCC patients

To prevent overfitting, we used the LASSO Cox regression analysis as shown in Figure 2C, selecting the optimum value (λ) by setting it equal to the vertical line based on the minimal criteria. As a result, an 18-ARG signature was constructed (Fig. 2C and D). The risk score of each patient was derived using the formula shown below:

Risk score = 0.059*EGFR-0.018*BMF + 0.084*CTTN + 0.093*BAK1 + 0.005*FADD-0.054*CDKN2A + 0.079*PLAU + 0.322*PLK1 + 0.490*PPARG-0.282*EZH2-0.018*CXCR4-0.046*LAMC2 + 0.285*SPINK1-0.159*SATB1-0.024*LTF + 0.106 *HSP90B1 + 0.123*TNFRSF12A + 0.055*SHC1. Similarly, the median risk score was employed in the classification of individuals with HNSCC in the GEO and TCGA datasets into high-risk and low-risk categories.

### 3.3. The landscape of ARGs in HNSCC patients

**Figure 3.**
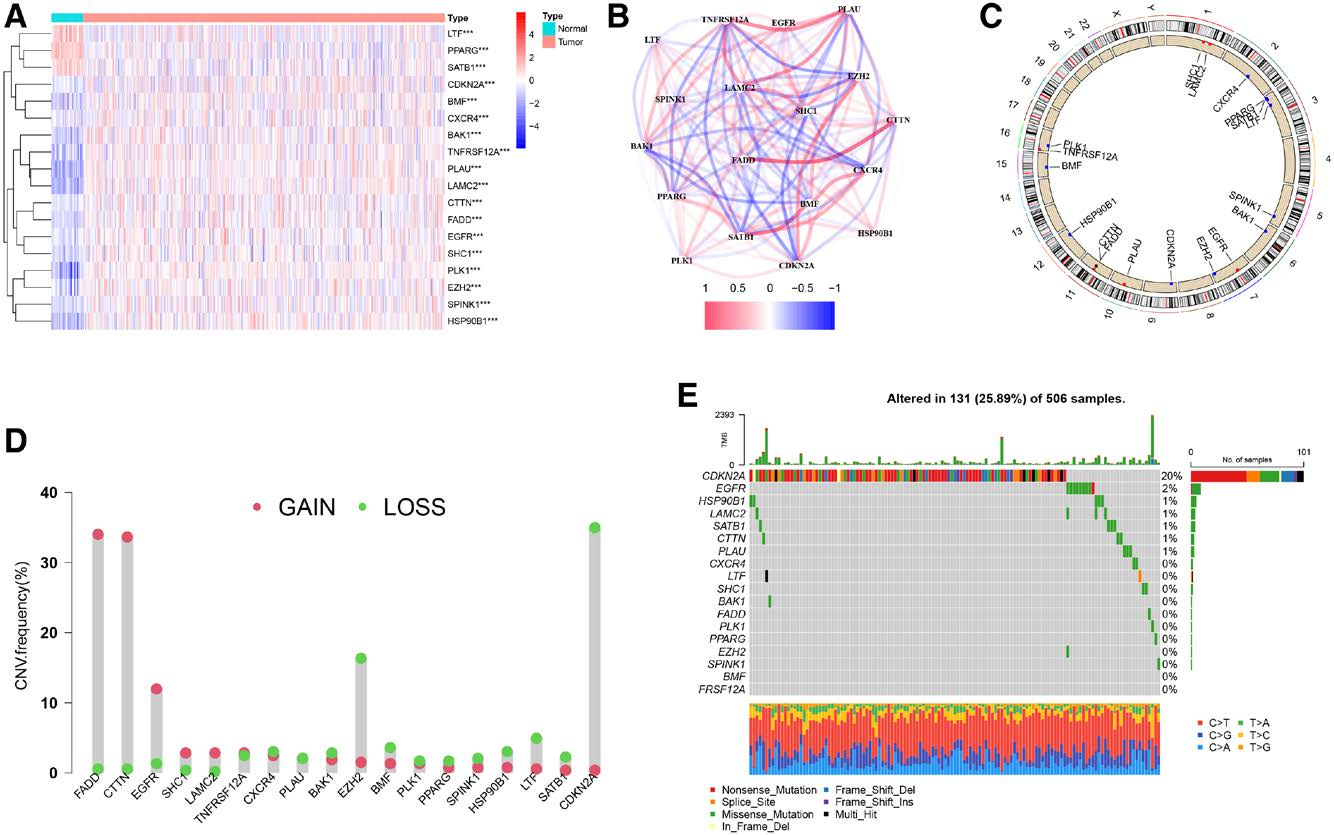
Landscape of ARGs expression, copy number variations, and mutation analysis in HNSCC patients. (A) A heatmap of the 18 ARGs between HNSCC tissues and neighboring healthy tissues. ***P < .001. (B) ARG’s association network (red depicts a positive association whereas blue depicts a negative association). (C) The locations of ARGs with CNV on chromosomes. (D) The CNV frequency of ARGs. (E) ARGs’ mutation frequency. Each patient is represented by a separate column. ARGs = anoikis-related genes, CNV = copy number variation, HNSCC = head and neck squamous carcinoma.

The heatmap of 18 ARGs expression was presented in Figure 3A. *LTF, PPARG*, and *SATB1* expression levels decreased in HNSCC tissues, otherwise, the other 15 AGRs were highly expressed in HNSCC. The correlation network of 18-ARGs was shown in Figure 3B. Further, we performed somatic mutations and CNV analysis to explore the genetic variation landscape of ARGs. The chromosomal location of ARGs with CNV alteration was shown in Figure 3C. And as shown in Figure 3D, *FADD* and *CTTN* had high amplification rates, while *EZH2* and *CDKN2A* had high rates of copy number deletions. For HNSCC patients, the overall mutation rate of ARGs was relatively low (approximately 25.89%). As displayed in Figure 3E, *CDKN2A* had a higher mutation rate of 20%, while other ARGs exhibited lower mutation frequencies in HNSCC samples.

### 3.4. Single-cell analysis

To explore the relationship between ARGs and TME in HNSCC, the single-cell sequencing dataset (GSE103322) was analyzed by TISCH. It demonstrated that cells were clustered into 20 clusters and 11 cell types, and the distribution and number of various cell types were displayed in Figure S1A–C (see Figure S1, Supplemental Digital Content, http://links.lww.com/MD/J567, which demonstrates the link between the ARGs and the TME). As shown in Figure S1D and E, ARGs were expressed in a variety of cells, including malignant cells, immune cells, and stromal cells. Specifically, *CTTN* was mainly detected in malignant cells and stromal cells (Endothelial, Fibroblasts, Myocyte, and Myofibroblasts). *CXCR4* was mainly expressed in immune cells (CD4Tconv, CD8T, CD8Tex, Mono/Macro, and Plasma). *LAMC2* was mainly found in malignant cells. *SPINK1* was almost only detected in Fibroblasts. *SHC1* and *HSP90B1* were expressed in many cell types, but *SHC1* was mainly expressed in malignant cells and stromal cells (Figure S1F and G).

### 3.5. Prognostic value of ARG signature

**Figure 4.**
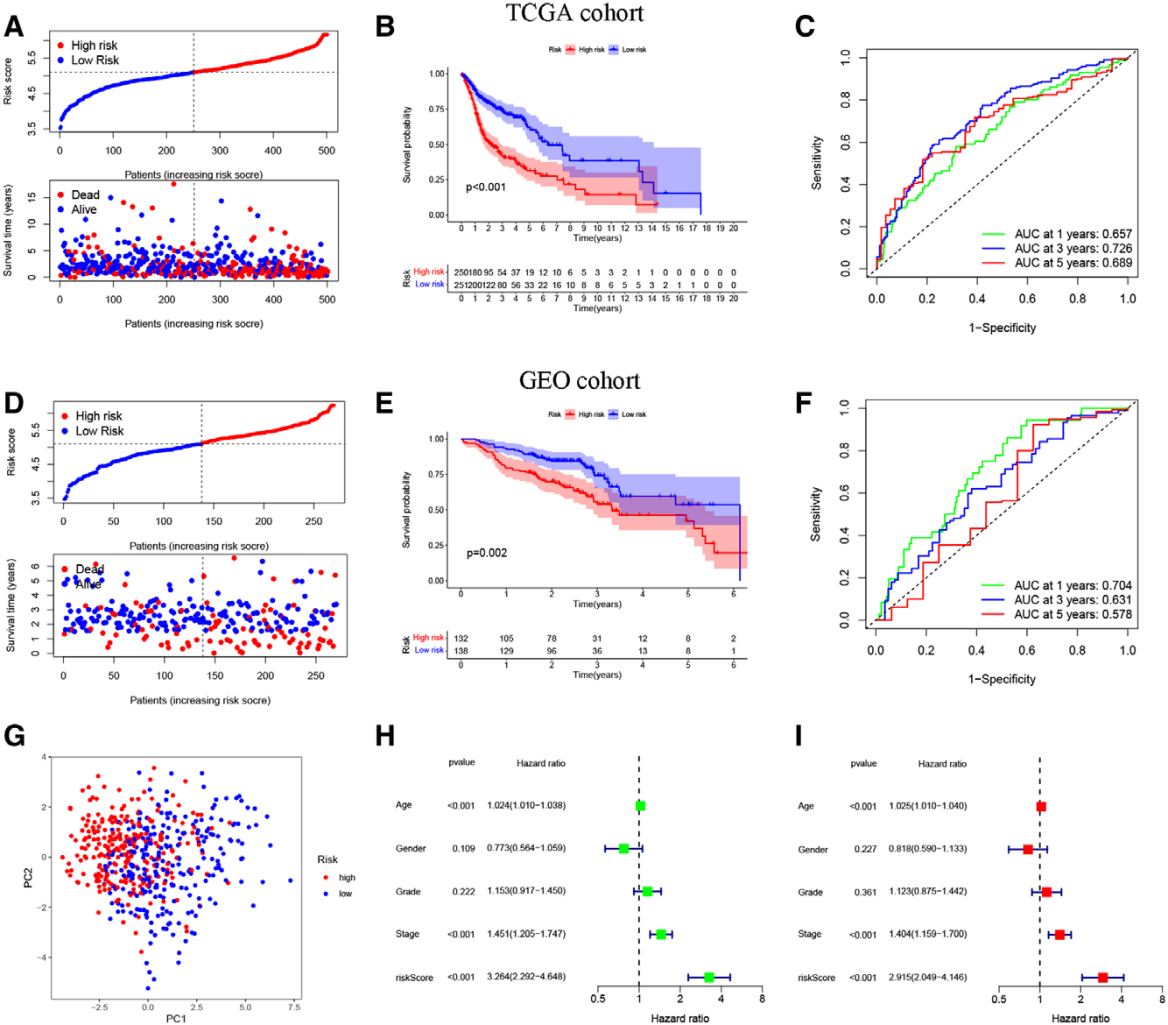
Independent prognostic value of ARG signature. (A) A scatter plot of the TCGA cohort’s OS status, OS time, and risk score distributions. (B) Kaplan–Meier survival curves for high-risk and low-risk HNSCC individuals in the TCGA cohort. (C) The AUC values of time-dependent ROC curves for OS prognosis in the TCGA category. (D) A scatter plot of the GEO cohort’s OS time, OS status, and risk score variations. (E) Kaplan–Meier survival curves of high-risk and low-risk HNSCC subjects in the GEO cohort. (F) The AUC values of time-dependent ROC curves for OS prognosis in the GEO cohort. (G) Score plot for the principal component analysis. (H) Evaluation of the clinicopathologic variables and risk score using univariate Cox regression analysis. (I) Multivariate Cox regression analysis of clinicopathologic characteristics and the risk score. ARGs = anoikis-related genes, AUC = area under the curve, GEO = gene expression omnibus, HNSCC = head and neck squamous carcinoma, OS = overall survival, ROC = receiver operating characteristic, TCGA = the cancer genome atlas.

**Figure 5.**
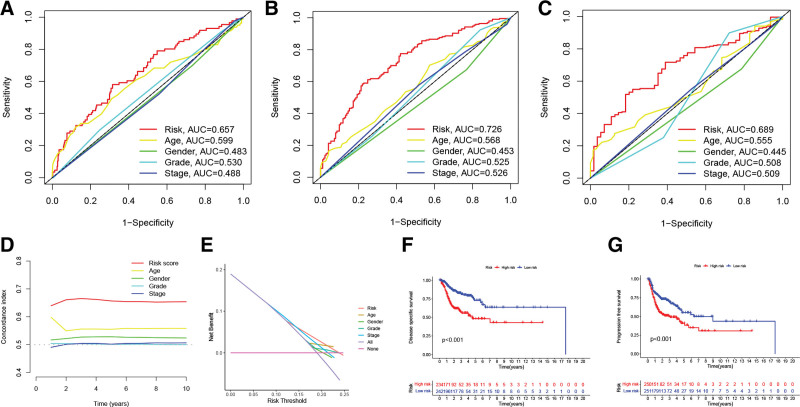
Predictive performance analysis of ARG signature. (A) The 1-year AUC values for clinicopathologic characteristics and risk scores for predicting OS. (B) The 3-year AUC values for clinicopathologic characteristics and risk scores for predicting OS. (C) The 5-year AUC values for clinicopathologic characteristics and risk scores for predicting OS. (D) The time dependents C-indexes of the risk score and clinicopathologic characteristics. (E) The decision curve analysis of the risk score and clinicopathologic characteristics. (F) Kaplan–Meier survival curves comparing high-risk and low-risk HNSCC patients’ disease-specific survival. (G) Kaplan–Meier survival curves comparing progression-free survival among individuals with low-risk and high-risk HNSCC. ARGs = anoikis-related genes, AUC = area under the curve, HNSCC = head and neck squamous carcinoma, OS = overall survival.

**Figure 6.**
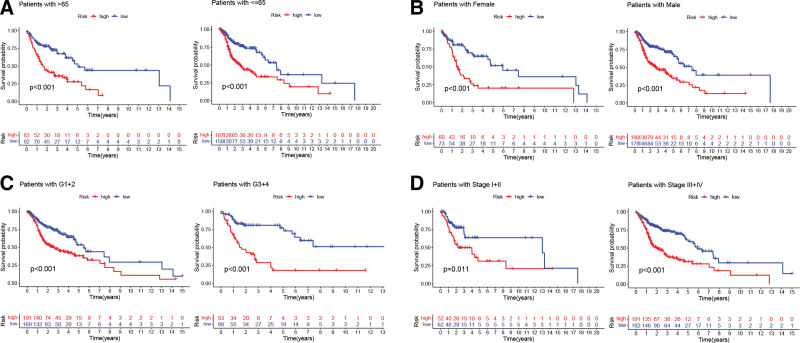
Subgroup survival analysis. Kaplan–Meier survival curves for age (A), gender (B), histologic grade (C), and clinical stage (D).

TCGA cohort was utilized as a training cohort, whereas the GEO cohort was employed as a validation cohort, to validate the ARG signature’s prognostic performance. Figure 4A shows the survival outcomes and risk score distributions for the TCGA cohort, revealing that greater mortality and shorter OS durations were observed among individuals with higher risk scores. Individuals in the high-risk group had shorter OS in contrast with ones in the low-risk group, as seen by TCGA cohort-related Kaplan–Meier curves (Fig. 4B). Figure 4C shows that our ARG signature had an excellent predictive performance for the prognostic outcomes of HNSCC patients over the course of 1, 3, and 5 years (AUC = 0.657, 0.726, and 0.689, respectively). Consistent with these findings, the validation cohort also found a strong association between reduced OS duration and high-risk patients (Fig. 4D–F). Furthermore, principal component analysis was conducted in the investigation of the potential of ARG signature to differentiate between the 2 categories (as shown in Fig. 4G). Results from further multivariate and univariate Cox regression analyses, shown in Figure 4H and I, respectively, corroborated the risk score’s role as an independent predictor of OS for patients diagnosed with HNSCC. More traditional clinical factors were also utilized in evaluating the risk score’s ability to predict outcomes. One-year (Fig. 5A), 3-year (Fig. 5B), and 5-year (Fig. 5C) OS prediction AUC values for the risk score were higher as compared to those for grade, age, gender, and stage, insinuating that the risk score may predict the OS of HNSCC subjects in a more accurate fashion. Age, grade, gender, and stage were not as effective as the ARG signature in predicting HNSCC prognosis, as shown by the time-dependent concordance indexes (Fig. 5D). The superior predictive performance of the ARG signature was further proven by a decision curve analysis (Fig. 5E). In addition, the Kaplan–Meier survival curves for HNSCC patients revealed that those with a low risk had a longer disease-specific survival (Fig. 5F) and progression-free survival (Fig. 5G). Clinical variables were also utilized in conducting a subgroup analysis. Individuals in the low-risk group exhibited a superior survival prognosis across all clinical subgroups such as age (Fig. 6A), gender (Fig. 6B), grade (Fig. 6C), and stage (Fig. 6D). Then, the ARG signature was compared with 5 previously published prognostic signatures^[21–25]^ in the TCGA-HNSC cohort to determine whether our ARG signature had a superior predictive performance (see Figure S2, Supplemental Digital Content, http://links.lww.com/MD/J568, which demonstrates the comparison of the ARG signature with other signatures). Although these 5 previously published prognostic signatures enabled the creation of 2 subgroups with significantly different prognoses (Figure S2A–E), the ROC curve analysis and RMST results indicated that our ARG signature was superior. The AUC values of the 5 signatures (Figure S2F–J) for 1-, 3-, and 5-year survival were lower than those of our ARG signature. Meanwhile, as calculated by RMST and compared with the other signatures, our signature had the highest C-index at 0.663 (Figure S2K and L).

### 3.6. Nomogram construction and validation for HNSCC individuals

**Figure 7.**
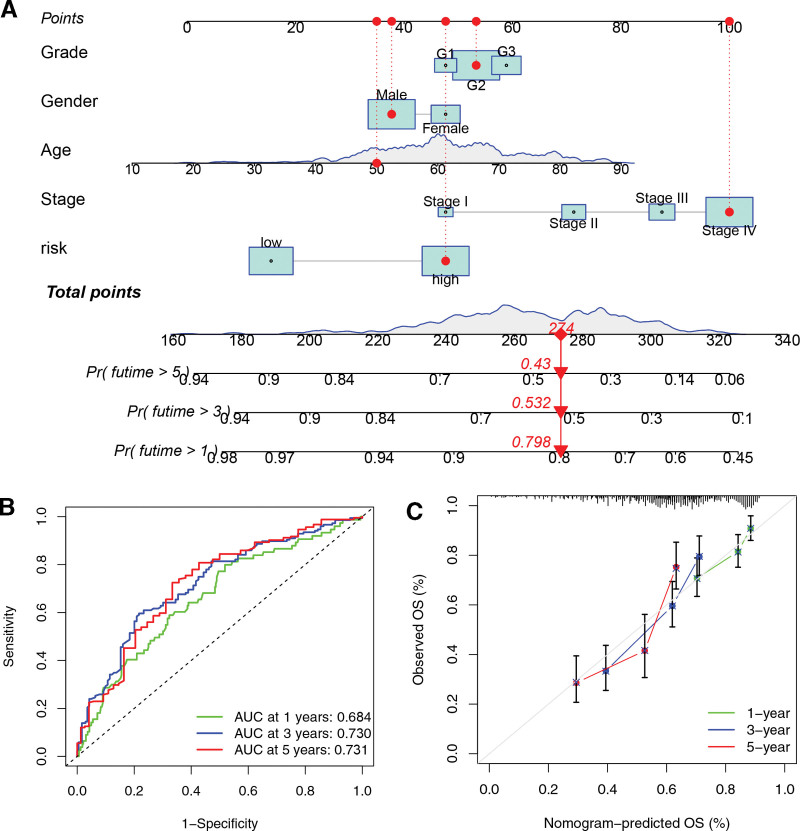
Development and verification of a nomogram for HNSCC individuals. (A) Nomogram for anticipating the 1-, 3-, or 5-year OS rate in HNSCC patients. (B) The nomogram’s ROC curves. (C) Nomogram calibration curves. HNSCC = head and neck squamous carcinoma, ROC = receiver operating characteristic.

A nomogram was developed using grade, gender, risk score, stage, and age to forecast the 1-, 3-, and 5-year survival (Fig. 7A). Accuracy was assessed by drawing ROC curves for the nomogram (Fig. 7B). The AUCs of the nomogram for 1-, 3-, and 5-year survival were 0.684, 0.730, and 0.731, respectively. These values were higher than those obtained from the clinical characteristic and risk score alone. Figure 7C demonstrates that the nomogram’s calibration curves were in close proximity to the standard line, suggesting that the nomogram performed well.

### 3.7. Relationships between clinical characteristics and risk score

**Figure 8.**
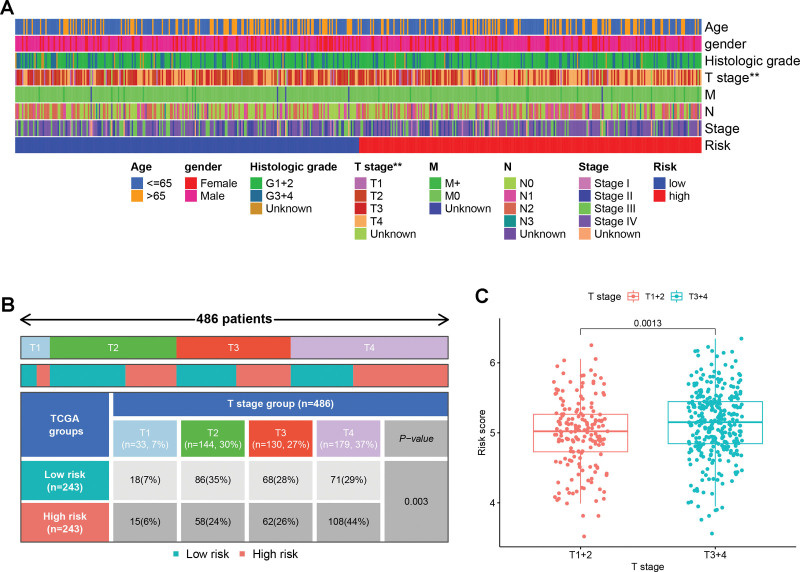
The link between the clinicopathological variables and the risk scores among HNSCC individuals. (A) A heatmap depicting the distribution of clinicopathological features of HNSCC patients into high-risk and low-risk categories. **P < .01. (B) A comparison of the T stage between the 2 categories was done via the chi-squared test. (C) The correlation between the T stage and risk scores was analyzed using the Wilcoxon signed-rank test. HNSCC = head and neck squamous carcinoma.

The correlation between clinical characteristics and the risk score was analyzed. The T stage was shown to have a statistically significant link to the risk score (Fig. 8A). Moreover, Figure 8B displays the outcomes of the chi-square test between the T stage and risk score, revealing a correlation between a higher risk score and an advanced T stage (*P* = .003). In line with this observation, the Wilcoxon signed-rank test revealed that individuals with HNSCC who were in advanced T stage had considerably higher risk scores than those who were in early T stage (Fig. 8C).

### 3.8. Relationships between TME and risk score in HNSCC

**Figure 9.**
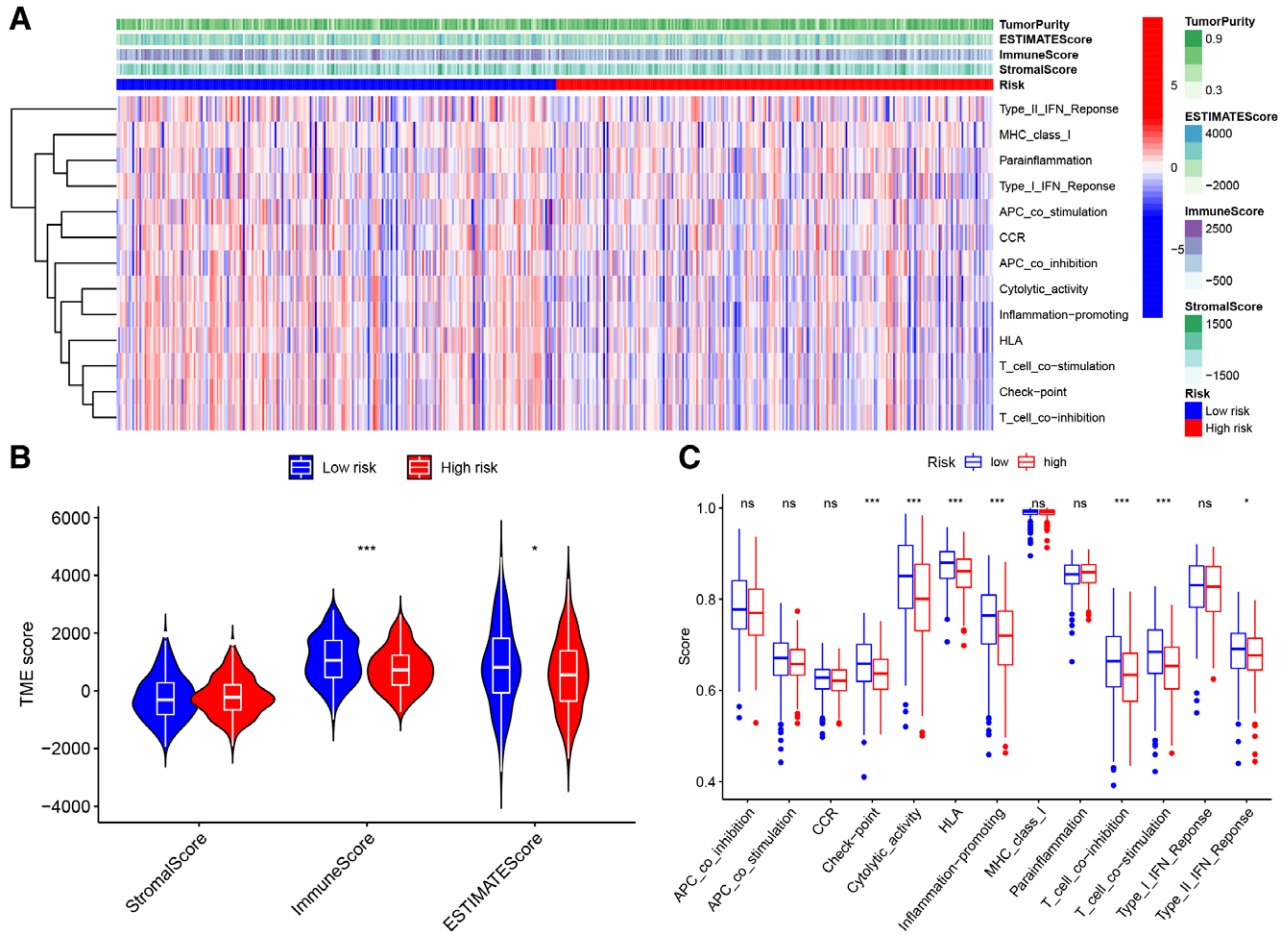
The risk scores’ effect on the TME in HNSCC individuals. (A) A heatmap depicting the relationship between the TME and the risk scores. (B) The immune, stromal, and ESTIMATE scores were calculated using the ESTIMATE method and compared between the 2 groups. (C) The low-risk group had higher ssGSEA scores for most immunological activities than the high-risk group. ***P < .001; **P < .01; *P < .05; ns = not significant. HNSCC = head and neck squamous carcinoma, ssGSEA = single-sample gene set enrichment analysis, TME = tumor microenvironment.

**Figure 10.**
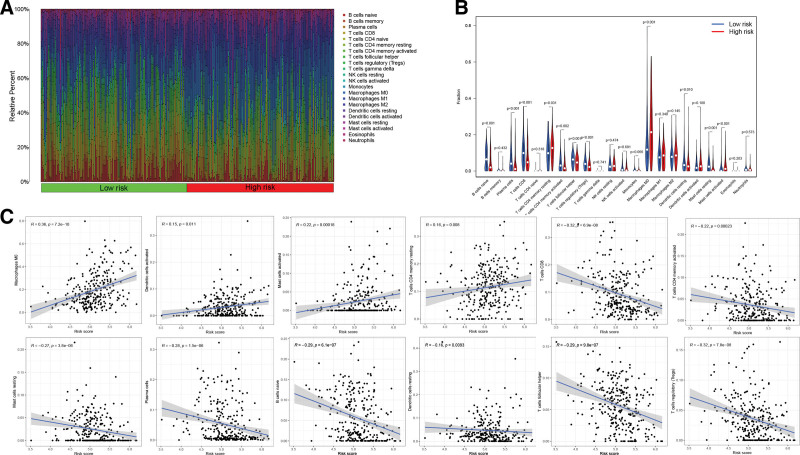
The association between risk score and infiltration status of the immune cells. (A) Individual HNSCC patients’ respective level of immune cell infiltration. (B) The violin plot comparing the infiltration levels of the immune cells between low-risk and high-risk categories. (C) The relationships between the risk score and the infiltration level of immune cells. HNSCC = head and neck squamous carcinoma.

The relationship between the TME and the risk score was illustrated in Figure 9A. Further analyses showed that low-risk HNSCC patients tended to have a higher level of immune infiltration and a larger proportion of immune components (Fig. 9B), whereas high-risk patients tended to have lower immune scores and ESTIMATE scores. In addition, 7 immune function scores were significantly poorer in the high-risk group compared to the low-risk group, including type II IFN response, checkpoint, T cell co-stimulation, HLA, cytolytic activity, T cell co-inhibition, and inflammation-promoting (Fig. 9C). Afterward, we conducted a CIBERSORT analysis to delve further into the correlation between risk score and the infiltration of immune cells (Fig. 10A). A negative link was found between the proportion of CD8^+^ T cells, activated CD4^+^ memory T cells, plasma cells, follicular helper T cells, resting mast cells, naive B cells, resting dendritic cells, and regulatory T cells, and risk score in the low-risk group. However, there was a significant relationship between the risk score and increased numbers of M0 macrophages, active mast cells, and resting CD4^+^ memory T cells in the high-risk group (Fig. 10B and C).

### 3.9. Immunotherapy prediction and drug sensitivity analysis

**Figure 11.**
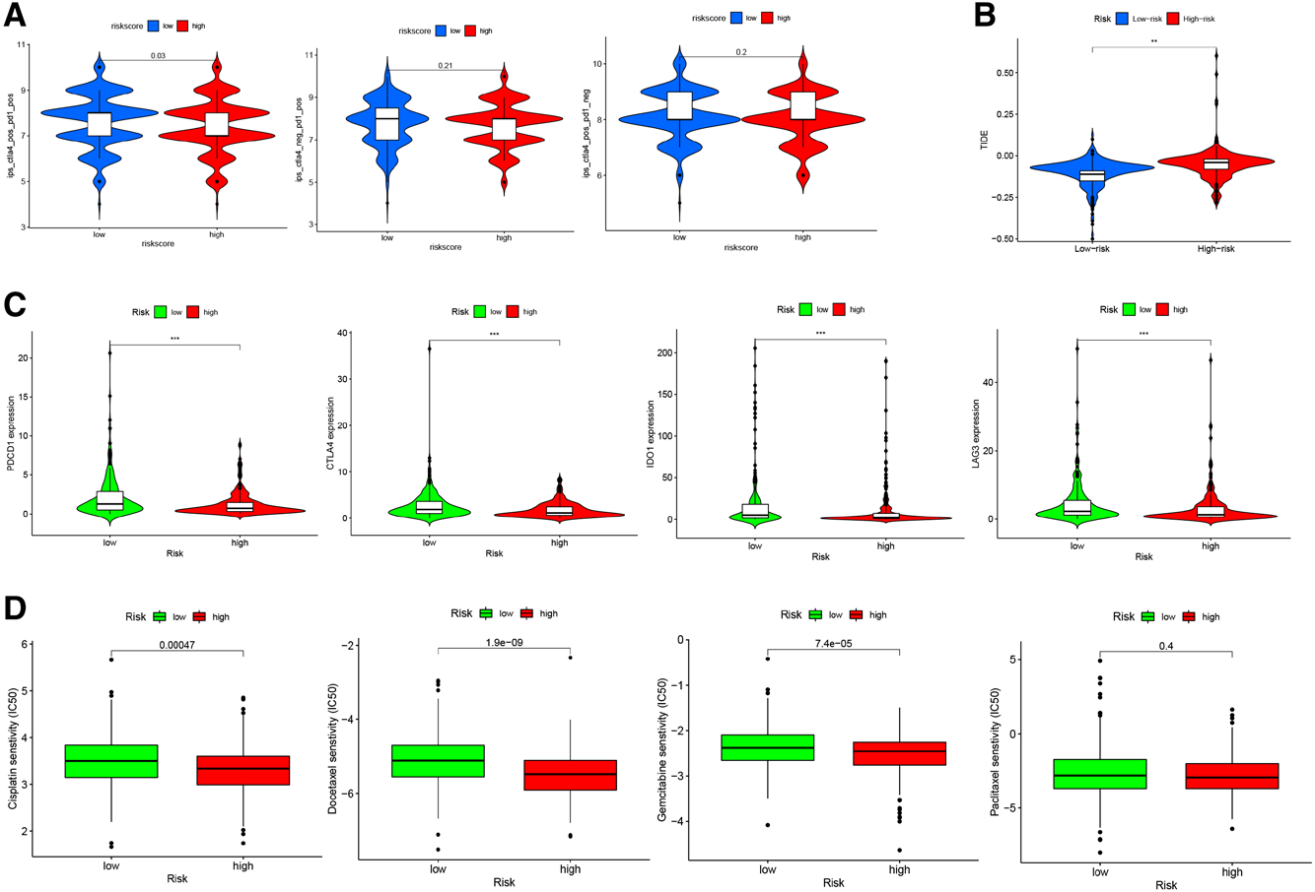
Immunotherapy prediction and drug sensitivity analysis for HNSCC patients in low-risk and high-risk groups. (A) Variations in IPS between individuals in low-risk and high-risk categories who received anti-CTLA4 and anti-PD1 combination treatment, anti-PD1 monotherapy, and anti-CTLA4 monotherapy. (B) Differences in TIDE scores between high-risk and low-risk categories. (C) The relationship between risk score and *PDCD1, CTLA4, IDO1*, and *LAG-3* expression. (D) The variations in the IC50 of cisplatin, docetaxel, gemcitabine, and paclitaxel between high-risk and low-risk categories. HNSCC = head and neck squamous carcinoma, IC50 = half maximal inhibitory concentration, IPS = immunophenoscores, TIDE = Tumor Immune Dysfunction and Exclusion.

The IPS was used to evaluate how well an individual was responding to immunotherapy, and higher IPS indicated more benefits from the immunotherapy. Figure 11A shows that subjects in the low-risk group had a trend toward a better reaction to combination treatment with anti-PD1 and anti-CTLA4 than the high-risk group. Nevertheless, no significant variations were identified between the 2 categories in anti-CTLA4 monotherapy or anti-PD1 monotherapy. In addition, the TIDE score was remarkably lower in the low-risk group than in the high-risk group (Fig. 11B). Patients with lower TIDE scores had a better response rate to immune checkpoint blockade (ICB) therapy because of the reduced risk of antitumor immune escape. After that, we assessed the relationship between the risk score and expression of ICB genes, including *PDCD1, CTLA4, IDO1*, and *LAG-3*. Notably, high-risk individuals had noticeably lower ICB gene expression compared to low-risk patients (Fig. 11C). Cisplatin, gemcitabine, docetaxel, and paclitaxel IC50 values were further investigated between the 2 groups. As can be seen in Figure 11D, patients in the high-risk group acquired better chemosensitivity, as shown by lower IC50 values for cisplatin, docetaxel, and gemcitabine compared to those in the low-risk group. However, no statistical difference was observed in the IC50 value for paclitaxel between the 2 categories.

### 3.10. Functional enrichment analysis

**Figure 12.**
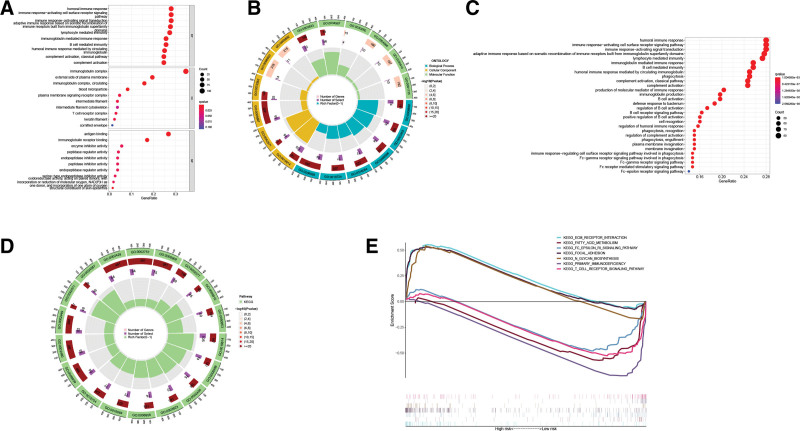
Functional enrichment analysis. (A) The top 10 MFs, BPs, and CCs from GO analysis of DEGs across high-risk and low-risk categories. (B) Circle plot for the GO enrichment analysis. (C) KEGG Enrichment analysis based on DEGs between high-risk and low-risk categories. (D) Circle plot for the KEGG enrichment analysis. (E) GSEA findings indicate 3 pathways that are enriched in the high-risk group and 4 pathways that are enriched in the low-risk group. BPs = biological process, CCs = cellular components, GO = gene ontology, GSEA = gene set enrichment analysis, MFs = molecular functions.

In addition, we employed the DEGs to conduct a gene ontology analysis of the differences between the low-risk and high-risk groups. Most DEGs were rich in immune-related biological processes including humoral immune response, activating signal transduction, etc (Fig. 12A and B). DEGs were also shown to be enriched in immune-related pathways, including humoral immune response, activating cell surface receptor signaling pathway, etc., according to the Kyoto Encyclopedia of Genes and Genomes analysis (Fig. 12C and D). In addition, the GSEA revealed enrichment of 3 ECM-related pathways in the high-risk group, namely ECM-receptor interaction, focal adhesion, and N-glycan biosynthesis, and 4 immune-related pathways in the low-risk group, including FC epsilon RI signaling pathway, T cell receptor signaling pathway, primary immunodeficiency, and fatty acid metabolism (Fig. 12E).

## 4. Discussion

The American Cancer Society placed HNSCC as the sixth most prevalent cancer, which constituted 5.3% to 7.1% of all systemic malignancies.^[29]^ In the case of HNSCC, metastasis is the leading contributor to treatment failure.^[30]^ As a result, it is crucial to come up with a prognostic model to promote the development of treatment methods for HNSCC. Anoikis, a mode of programmed cell death triggered by cell detachment from the ECM, plays an important part in development, organism homeostasis, illness, and tumor spread by limiting adherent-independent cell growth and attachment to an inappropriate matrix.^[31,32]^ When cells no longer have touch with the ECM, the programmed cell death anoikis is triggered. Nonetheless, tumor cells devise a number of coping mechanisms to resist anoikis, such as postponing the onset of anoikis via mutual phagocytosis and autophagy and increasing the percentage of metastatic cells that survive.^[15]^ Tumor cells’ ability to develop anoikis resistance is crucial to their ability to survive while in circulation, a condition essential for metastasis.^[31,33]^ Anoikis has been linked to tumor growth and treatment failure, so it’s no surprise that it’s a major player in the metastasis of lung cancer.^[34]^ Inducing Bit1-mediated anoikis, as shown by Brunquell et al,^[35]^ may be a successful method of treating breast cancer. STAT3 enhanced anoikis resistance, facilitated cell invasion, and enhanced pancreatic cancer cells’ capacity to metastasize, as reported by Fofaria and Srivastava.^[36]^ Researchers have identified some prognostic signatures for HNSCC in recent years.^[21–25,37]^ However, to our knowledge, few have investigated the relationship between anoikis and HNSCC. Here, we established a new anoikis-related prognostic signature by identifying 18 ARGs with prognostic significance. Compared with 5 other signatures,^[21–25]^ our model showed better predictive efficacy in predicting the prognosis of HNSCC patients. Moreover, we found that our model had potential ability to predict the sensitivity to chemotherapy and immunotherapy, which may suggest immunotherapeutic and chemotherapeutic strategies for patients with HNSCC.

Given the heterogeneity of HNSCC tumor cells,^[38,39]^ we evaluated amplification rates of ARGs and discovered that *FADD* and *CTTN* were often amplified in HNSCC tumor cells. The FADD protein was an adapter molecule that facilitated apoptotic signals via the mechanism of interacting with many different types of cell surface receptors.^[40]^ According to our univariate Cox regression analysis, greater *FADD* expression in patients with HNSCC was related to worse survival rates, which is in line with prior findings.^[41]^ It has been reported that *CTTN*, a substrate for SRC, induced gefitinib resistance in HNSCC.^[42]^ Also, it has been found that phosphorylation of *CTTN* promotes metastasis and cell motility.^[43]^ Mutation and copy number deletions were also observed to be significantly higher in *CDKN2A. CDKN2A* was considered a tumor suppressor gene.^[44]^ Overexpression of *CDKN2A* in HNSCC has been linked to improved survival, and studies have shown that deletions of *CDKN2A* are related to susceptibility to checkpoint inhibitors.^[45–47]^ Together, the aforementioned ARGs had substantial impacts on HSNCC patients’ prognoses. Furthermore, single-cell analysis indicated the heterogeneity of the HNSCC TME and may help to inform future immunotherapy interventions.

According to our analysis, this prognostic signature divided HNSCC patients into high- and low-risk groups, similar to the studies in glioblastoma,^[17,48]^ high-risk group individuals generally having a worse prognosis than individuals in the low-risk group. Cox regression analysis also suggested the risk score as an independent risk predictor for OS in HNSCC patients. The risk scores that were based on the prognostic signature had substantially higher 1-, 3-, and 5-year AUC values, indicating superior prognostic effects in HNSCC patients, compared to other clinical features. Subgroup analysis by gender, age, clinical stage, and histologic grade confirmed the signature’s applicability across all subgroups, further highlighting the signature’s universal applicability. As a prognostic model, the nomogram was also extensively employed in clinical settings.^[49,50]^ Modeling the nomogram with the use of risk scores and clinical characteristics, the calibration and ROC curves depicted that the nomogram was accurate and had a strong predictive performance. This signature was shown to be reliable and effective in predicting the prognosis of patients with HNSCC, as evidenced by the aforementioned findings.

Treatment with immunotherapy shows promise for those with HNSCC.^[51]^ Despite new research showing the TME’s importance in immunotherapy,^[52,53]^ the TME of HNSCC remained heterogeneous and diverse.^[54]^ Improving the efficacy of immunotherapy requires further investigation into the involvement of TME. The correlation between the risk score and TME was analyzed in this study. We discovered that patients classified as low-risk had higher immune scores, which is indicative of more infiltration by immune cells. A similar pattern was seen using the single-sample GSEA method, which identified the enrichment of 7 immunological pathways in the low-risk group. Immune cells including, neutrophils, B cells, and CD8^+^ T cells, were shown to be more plentiful in the tissue and blood of HNSCC subjects with a positive prognosis.^[55]^ CIBERSORT studies found low infiltration levels of CD8^+^ T cells and naive B cells in high-risk individuals, which may be a factor in their worse prognosis. Moreover, it is possible that resting CD4^+^ memory T cells blocked CD8^+^ T cell activation and NK cell activity, hence suppressing the immunological response.^[56]^ Specifically, Wang et al^[57]^ found a positive correlation between the number of CD4^+^ T cells and the number of active CD4^+^ memory T cells, but a negative correlation with resting CD4^+^ memory T cells. In this study, high-risk patients had a higher infiltration level of resting CD4^+^ memory T cells but a lower infiltration level of activated CD4^+^ memory T cells and CD8^+^ T cells in comparison to low-risk patients. This evidence insinuates that the high-risk population may fare worse as a consequence of immune evasion and dysfunction, as discussed above.

Immunotherapy has come a long way in recent years, and ICBs like anti-PD-1/PD-L1 antibodies have completely changed how HNSCC is treated.^[58]^ However, the majority of HNSCC patients were resistant to PD-1 blocking, and the response rate was only 20% to 30%.^[59]^ Therefore, it is crucial to anticipate patients’ response to immunotherapy for the purpose of developing better treatment options. The goal of this study was to see whether the ARGs signature is connected to a positive immunotherapy response among individuals with HNSCC. We demonstrated that ICB-related gene expression (*CTLA4, PDCD1, LAG-3*, and *IDO1*) was considerably greater in individuals in the low-risk group compared to the ones in the high-risk group, insinuating that the individuals in the low-risk group would have a better reaction to ICB therapy. We discovered that the TIDE score supported our original hypothesis that individuals from the low-risk group responded in a better fashion to ICB treatment than individuals from the high-risk group. Moreover, the IPS results showed that individuals from the high-risk group responded less favorably to combination therapy with anti-PD1 and anti-CTLA4 as compared to individuals from the low-risk group. Altogether, these results suggest that the ARG signature developed in this work has the potential to act as a predictive marker for immunotherapy and might lead to the development of more accurate immunotherapy schemes.

In addition, chemotherapy had been an essential part of comprehensive therapies for HNSCC patients, which had been shown to increase survival rates for those diagnosed with the disease.^[60,61]^ Nonetheless, cisplatin resistance has emerged as a significant barrier to curative therapy for HNSCC.^[62]^ Four commonly used chemotherapeutic drugs for HNSCC treatment had their IC50 values examined in the present investigation. Treatment with docetaxel, cisplatin, and gemcitabine was significantly more sensitive in the high-risk group as compared to the low-risk group.

It’s important to note that there are limitations to this research. This study’s findings relied only on data from TCGA and GEO, and it contained a small sample size of patients. Our conclusions would have greater credibility if they were supported by further experiments. Further research is needed to explore the mechanism of the anoikis-based prognostic model’s effect on the immunotherapy of HNSCC.

## 5. Conclusion

As a result of this investigation, a novel ARG signature was created for use in predicting the prognosis of HNSCC. We discovered that patients with HNSCC who had lower risk scores responded better to combination immunotherapy and had a better prognosis, whereas individuals in the high-risk group responded better to chemotherapy. Our research might help in tailoring treatments for people with HNSCC.

## Acknowledgments

We thank all the R programming package developers.

## Author contributions

**Conceptualization:** Chongchang Zhou, Hongxia Deng.

**Funding acquisition:** Zhisen Shen.

**Software:** Chongchang Zhou, Zhisen Shen.

**Supervision:** Zhisen Shen.

**Validation:** Yi Shen.

**Writing – original draft:** Zhengyu Wei.

**Writing – review & editing:** Chongchang Zhou.

## Supplementary Material





## References

[1] Gauthier A Philouze P Lauret A. Circulating tumor cell detection during neoadjuvant chemotherapy to predict early response in locally advanced oropharyngeal cancers: a prospective pilot study. J Pers Med. 2022;12:445. 35330447 10.3390/jpm12030445 PMC8950569

[2] Kostrzewska-Poczekaj M Bednarek K Jarmuz-Szymczak M. Copy number gains of the putative CRKL oncogene in laryngeal squamous cell carcinoma result in strong nuclear expression of the protein and influence cell proliferation and migration. Sci Rep. 2020;10:24. 31913340 10.1038/s41598-019-56870-5 PMC6949282

[3] Liu Z Zhang D Liu C. Comprehensive analysis of myeloid signature genes in head and neck squamous cell carcinoma to predict the prognosis and immune infiltration. Front Immunol. 2021;12:659184. 33995379 10.3389/fimmu.2021.659184 PMC8116959

[4] Qi Z Qiu Y Wang Z. A novel diphtheria toxin-based bivalent human EGF fusion toxin for treatment of head and neck squamous cell carcinoma. Mol Oncol. 2021;15:1054–68. 33540470 10.1002/1878-0261.12919 PMC8024719

[5] Lechner A Schlößer HA Thelen M. Tumor-associated B cells and humoral immune response in head and neck squamous cell carcinoma. Oncoimmunology. 2019;8:1535293. 30723574 10.1080/2162402X.2018.1535293 PMC6350680

[6] Wang L Chen C Li F. Overexpression of neuromedin U is correlated with regional metastasis of head and neck squamous cell carcinoma. Mol Med Rep. 2016;14:1075–82. 27279246 10.3892/mmr.2016.5347 PMC4940074

[7] Meng HX Miao SS Chen K. Association of p16 as prognostic factors for oropharyngeal cancer: evaluation of p16 in 1470 patients for a 16 year study in Northeast China. Biomed Res Int. 2018;2018:9594568. 30310820 10.1155/2018/9594568 PMC6166388

[8] Xing L Zhang X Zhang X. Expression scoring of a small-nucleolar-RNA signature identified by machine learning serves as a prognostic predictor for head and neck cancer. J Cell Physiol. 2020;235:8071–84. 31943178 10.1002/jcp.29462 PMC7540035

[9] Lyu H Li M Jiang Z. Correlate the TP53 mutation and the HRAS mutation with immune signatures in head and neck squamous cell cancer. Comput Struct Biotechnol J. 2019;17:1020–30. 31428295 10.1016/j.csbj.2019.07.009 PMC6695281

[10] McLaughlin M Barker HE Khan AA. HSP90 inhibition sensitizes head and neck cancer to platin-based chemoradiotherapy by modulation of the DNA damage response resulting in chromosomal fragmentation. BMC Cancer. 2017;17:86. 28143445 10.1186/s12885-017-3084-0 PMC5282703

[11] Misawa K Mima M Satoshi Y. Neuropeptide receptor genes GHSR and NMUR1 are candidate epigenetic biomarkers and predictors for surgically treated patients with oropharyngeal cancer. Sci Rep. 2020;10:1007. 31974445 10.1038/s41598-020-57920-z PMC6978330

[12] Xu T Qin L Zhu Z. MicroRNA-31 functions as a tumor suppressor and increases sensitivity to mitomycin-C in urothelial bladder cancer by targeting integrin α5. Oncotarget. 2016;7:27445–57. 27050274 10.18632/oncotarget.8479 PMC5053662

[13] Griffiths GS Grundl M Leychenko A. Bit-1 mediates integrin-dependent cell survival through activation of the NFkappaB pathway. J Biol Chem. 2011;286:14713–23. 21383007 10.1074/jbc.M111.228387 PMC3077668

[14] Zhang X Xu LH Yu Q. Cell aggregation induces phosphorylation of PECAM-1 and Pyk2 and promotes tumor cell anchorage-independent growth. Mol Cancer. 2010;9:7. 20074345 10.1186/1476-4598-9-7 PMC2820017

[15] Guadamillas MC Cerezo A Del Pozo MA. Overcoming anoikis – pathways to anchorage-independent growth in cancer. J Cell Sci. 2011;124:3189–97. 21940791 10.1242/jcs.072165

[16] Du S Miao J Zhu Z. NADPH oxidase 4 regulates anoikis resistance of gastric cancer cells through the generation of reactive oxygen species and the induction of EGFR. Cell Death Dis. 2018;9:948. 30237423 10.1038/s41419-018-0953-7 PMC6148243

[17] Zhao S Chi H Ji W. A bioinformatics-based analysis of an anoikis-related gene signature predicts the prognosis of patients with low-grade gliomas. Brain Sci. 2022;12:1349. 36291283 10.3390/brainsci12101349 PMC9599312

[18] Guo C Xu LF Li HM. Transcriptomic study of the mechanism of anoikis resistance in head and neck squamous carcinoma. PeerJ. 2019;7:e6978. 31198634 10.7717/peerj.6978 PMC6535219

[19] Peltanova B Raudenska M Masarik M. Effect of tumor microenvironment on pathogenesis of the head and neck squamous cell carcinoma: a systematic review. Mol Cancer. 2019;18:63. 30927923 10.1186/s12943-019-0983-5 PMC6441173

[20] Puram SV Tirosh I Parikh AS. Single-cell transcriptomic analysis of primary and metastatic tumor ecosystems in head and neck cancer. Cell. 2017;171:1611–1624.e24. 29198524 10.1016/j.cell.2017.10.044 PMC5878932

[21] Jiang X Ke J Jia L. A novel cuproptosis-related gene signature of prognosis and immune microenvironment in head and neck squamous cell carcinoma cancer. J Cancer Res Clin Oncol. 2023;149:203–18. 36376617 10.1007/s00432-022-04471-7 PMC11798322

[22] Ming R Li X Wang E. The prognostic signature of head and neck squamous cell carcinoma constructed by immune-related RNA-binding proteins. Front Oncol. 2022;12:795781. 35449571 10.3389/fonc.2022.795781 PMC9016149

[23] Yang C Mei H Peng L. Prognostic correlation of an autophagy-related gene signature in patients with head and neck squamous cell carcinoma. Comput Math Methods Med. 2020;2020:7397132. 33456497 10.1155/2020/7397132 PMC7785385

[24] Zhang S Zhang W Zhang J. 8-Gene signature related to CD8(+) T cell infiltration by integrating single-cell and bulk RNA-sequencing in head and neck squamous cell carcinoma. Front Genet. 2022;13:938611. 35938006 10.3389/fgene.2022.938611 PMC9355512

[25] Zhao H Wang F Wang X. HPV-related prognostic signature predicts survival in head and neck squamous cell carcinoma. J Oncol. 2022;2022:7357566. 36425940 10.1155/2022/7357566 PMC9681561

[26] Yoshihara K Shahmoradgoli M Martínez E. Inferring tumour purity and stromal and immune cell admixture from expression data. Nat Commun. 2013;4:2612. 24113773 10.1038/ncomms3612 PMC3826632

[27] Newman AM Liu CL Green MR. Robust enumeration of cell subsets from tissue expression profiles. Nat Methods. 2015;12:453–7. 25822800 10.1038/nmeth.3337 PMC4739640

[28] Zhou C Shen Y Jin Y. A novel Pyroptosis-related long non-coding RNA signature for predicting the prognosis and immune landscape of head and neck squamous cell carcinoma. Cancer Med. 2022;11:5097–112. 35567376 10.1002/cam4.4819 PMC9761069

[29] Li Y Pan M Lu T. RAF1 promotes lymphatic metastasis of hypopharyngeal carcinoma via regulating LAGE1: an experimental research. J Transl Med. 2022;20:255. 35668458 10.1186/s12967-022-03468-7 PMC9172115

[30] Zhang J Lin H Jiang H. A key genomic signature associated with lymphovascular invasion in head and neck squamous cell carcinoma. BMC Cancer. 2020;20:266. 32228488 10.1186/s12885-020-06728-1 PMC7106876

[31] Mo H Guan J Mo L. ATF4 regulated by MYC has an important function in anoikis resistance in human osteosarcoma cells. Mol Med Rep. 2018;17:3658–66. 29257326 10.3892/mmr.2017.8296 PMC5802171

[32] Galardi A Colletti M Lavarello C. Proteomic profiling of retinoblastoma-derived exosomes reveals potential biomarkers of vitreous seeding. Cancers (Basel). 2020;12:1555. 32545553 10.3390/cancers12061555 PMC7352325

[33] Jin L Chun J Pan C. The PLAG1-GDH1 axis promotes anoikis resistance and tumor metastasis through CamKK2-AMPK signaling in LKB1-deficient lung cancer. Mol Cell. 2018;69:87–99.e7. 29249655 10.1016/j.molcel.2017.11.025 PMC5777230

[34] Wang J Luo Z Lin L. Anoikis-associated lung cancer metastasis: mechanisms and therapies. Cancers (Basel). 2022;14:4791. 36230714 10.3390/cancers14194791 PMC9564242

[35] Brunquell C Biliran H Jennings S. TLE1 is an anoikis regulator and is downregulated by Bit1 in breast cancer cells. Mol Cancer Res. 2012;10:1482–95. 22952044 10.1158/1541-7786.MCR-12-0144 PMC3651916

[36] Fofaria NM Srivastava SK. STAT3 induces anoikis resistance, promotes cell invasion and metastatic potential in pancreatic cancer cells. Carcinogenesis. 2015;36:142–50. 25411359 10.1093/carcin/bgu233 PMC4291051

[37] Qian X Tang J Chu Y. A novel pyroptosis-related gene signature for prognostic prediction of head and neck squamous cell carcinoma. Int J Gen Med. 2021;14:7669–79. 34764680 10.2147/IJGM.S337089 PMC8575318

[38] Tong M Han BB Holpuch AS. Inherent phenotypic plasticity facilitates progression of head and neck cancer: endotheliod characteristics enable angiogenesis and invasion. Exp Cell Res. 2013;319:1028–42. 23370231 10.1016/j.yexcr.2013.01.013 PMC3602379

[39] Suh Y Amelio I Guerrero Urbano T. Clinical update on cancer: molecular oncology of head and neck cancer. Cell Death Dis. 2014;5:e1018. 24457962 10.1038/cddis.2013.548 PMC4040714

[40] Grunert M Gottschalk K Kapahnke J. The adaptor protein FADD and the initiator caspase-8 mediate activation of NF-κB by TRAIL. Cell Death Dis. 2012;3:e414. 23096115 10.1038/cddis.2012.154 PMC3481141

[41] Li C Wu ZH Yuan K. Autophagy-related signature for head and neck squamous cell carcinoma. Dis Markers. 2020;2020:8899337. 33133307 10.1155/2020/8899337 PMC7591969

[42] Solanki HS Raja R Zhavoronkov A. Targeting focal adhesion kinase overcomes erlotinib resistance in smoke induced lung cancer by altering phosphorylation of epidermal growth factor receptor. Oncoscience. 2018;5:21–38. 29556515 10.18632/oncoscience.395 PMC5854290

[43] Zhu L Cho E Zhao G. The pathogenic effect of cortactin tyrosine phosphorylation in cutaneous squamous cell carcinoma. In Vivo. 2019;33:393–400. 30804117 10.21873/invivo.11486 PMC6506328

[44] Oikawa Y Morita KI Kayamori K. Receptor tyrosine kinase amplification is predictive of distant metastasis in patients with oral squamous cell carcinoma. Cancer Sci. 2017;108:256–66. 27889930 10.1111/cas.13126 PMC5329163

[45] Pérez Sayáns M Chamorro Petronacci CM Lorenzo Pouso AI. Comprehensive genomic review of TCGA head and neck squamous cell carcinomas (HNSCC). J Clin Med. 2019;8:1896. 31703248 10.3390/jcm8111896 PMC6912350

[46] Kagiyama Y Fujita S Shima Y. CDKN1C-mediated growth inhibition by an EZH1/2 dual inhibitor overcomes resistance of mantle cell lymphoma to ibrutinib. Cancer Sci. 2021;112:2314–24. 33792119 10.1111/cas.14905 PMC8177787

[47] Gadhikar MA Zhang J Shen L. CDKN2A/p16 deletion in head and neck cancer cells is associated with CDK2 activation, replication stress, and vulnerability to CHK1 inhibition. Cancer Res. 2018;78:781–97. 29229598 10.1158/0008-5472.CAN-17-2802 PMC5811346

[48] Sun Z Zhao Y Wei Y. Identification and validation of an anoikis-associated gene signature to predict clinical character, stemness, IDH mutation, and immune filtration in glioblastoma. Front Immunol. 2022;13:939523. 36091049 10.3389/fimmu.2022.939523 PMC9452727

[49] Zou W Zhu C Wang Z. A novel nomogram based on log odds of metastatic lymph nodes to predict overall survival in patients with perihilar cholangiocarcinoma after surgery. Front Oncol. 2021;11:649699. 34367951 10.3389/fonc.2021.649699 PMC8340771

[50] Wang M Liu W Xu Y. Predicting bleeding risk in a Chinese immune thrombocytopenia (ITP) population: development and assessment of a new predictive nomogram. Sci Rep. 2020;10:15337. 32948823 10.1038/s41598-020-72275-1 PMC7501260

[51] Mei Z Huang J Qiao B. Immune checkpoint pathways in immunotherapy for head and neck squamous cell carcinoma. Int J Oral Sci. 2020;12:16. 32461587 10.1038/s41368-020-0084-8 PMC7253444

[52] Frankel T Lanfranca MP Zou W. The role of tumor microenvironment in cancer immunotherapy. Adv Exp Med Biol. 2017;1036:51–64. 29275464 10.1007/978-3-319-67577-0_4

[53] Pitt JM Marabelle A Eggermont A. Targeting the tumor microenvironment: removing obstruction to anticancer immune responses and immunotherapy. Ann Oncol. 2016;27:1482–92. 27069014 10.1093/annonc/mdw168

[54] Xing DT Khor R Gan H. Recent research on combination of radiotherapy with targeted therapy or immunotherapy in head and neck squamous cell carcinoma: a review for radiation oncologists. Cancers (Basel). 2021;13:5716. 34830871 10.3390/cancers13225716 PMC8616456

[55] Cui J Zheng L Zhang Y. Bioinformatics analysis of DNMT1 expression and its role in head and neck squamous cell carcinoma prognosis. Sci Rep. 2021;11:2267. 33500531 10.1038/s41598-021-81971-5 PMC7838186

[56] Yuan W Cai W Huang X. Prognostic value of immune scores in the microenvironment of colorectal cancer. Oncol Lett. 2020;20:256. 32994819 10.3892/ol.2020.12119 PMC7509622

[57] Wang Y Ba HJ Liu ZC. Prognostic value of immune cell infiltration in bladder cancer: a gene expression-based study. Oncol Lett. 2020;20:1677–84. 32724410 10.3892/ol.2020.11750 PMC7377040

[58] Burcher KM Lantz JW Gavrila E. Relationship between tumor mutational burden, PD-L1, patient characteristics, and response to immune checkpoint inhibitors in head and neck squamous cell carcinoma. Cancers (Basel). 2021;13:5733. 34830888 10.3390/cancers13225733 PMC8616373

[59] Jia L Wang Y Wang CY. circFAT1 promotes cancer stemness and immune evasion by promoting STAT3 activation. Adv Sci (Weinh). 2021;8:2003376. 34258151 10.1002/advs.202003376 PMC8261519

[60] Hasegawa Y Goto M Hanai N. Predictive biomarkers for combined chemotherapy with 5-fluorouracil and cisplatin in oro- and hypopharyngeal cancers. Mol Clin Oncol. 2018;8:378–86. 29399358 10.3892/mco.2017.1521 PMC5774537

[61] Mei M Chen YH Meng T. Comparative efficacy and safety of radiotherapy/cetuximab versus radiotherapy/chemotherapy for locally advanced head and neck squamous cell carcinoma patients: a systematic review of published, primarily non-randomized, data. Ther Adv Med Oncol. 2020;12:1758835920975355. 10.1177/1758835920975355 PMC7727048 33343720

[62] Dong J Li J Li Y. Transcriptional super-enhancers control cancer stemness and metastasis genes in squamous cell carcinoma. Nat Commun. 2021;12:3974. 34172737 10.1038/s41467-021-24137-1 PMC8233332

