# Supplementary material for: Identification of a new anoikis-related gene signature for prognostic significance in head and neck squamous carcinomas
Source: Medicine (Baltimore). 2023 Sep 8;102(36):e34790. doi: 10.1097/MD.0000000000034790 (PMC10489427; doi:10.1097/MD.0000000000034790)
Supplement: Supplementary file 1 [file medi-102-e34790-s001.pdf]

Figure S1: The link between the ARGs and the tumor immune microenvironment. (A) The UMAP plot with cells colored by cluster ID. (B) The UMAP plot with cells colored by cell type. (C) The cell type proportion of each sample. (D) The expression of ARGs. (E) Expression of ARGs in each cell type. (F) The expression of each gene of ARG signature. (G) Expression of each gene of ARG signature in each cell type.

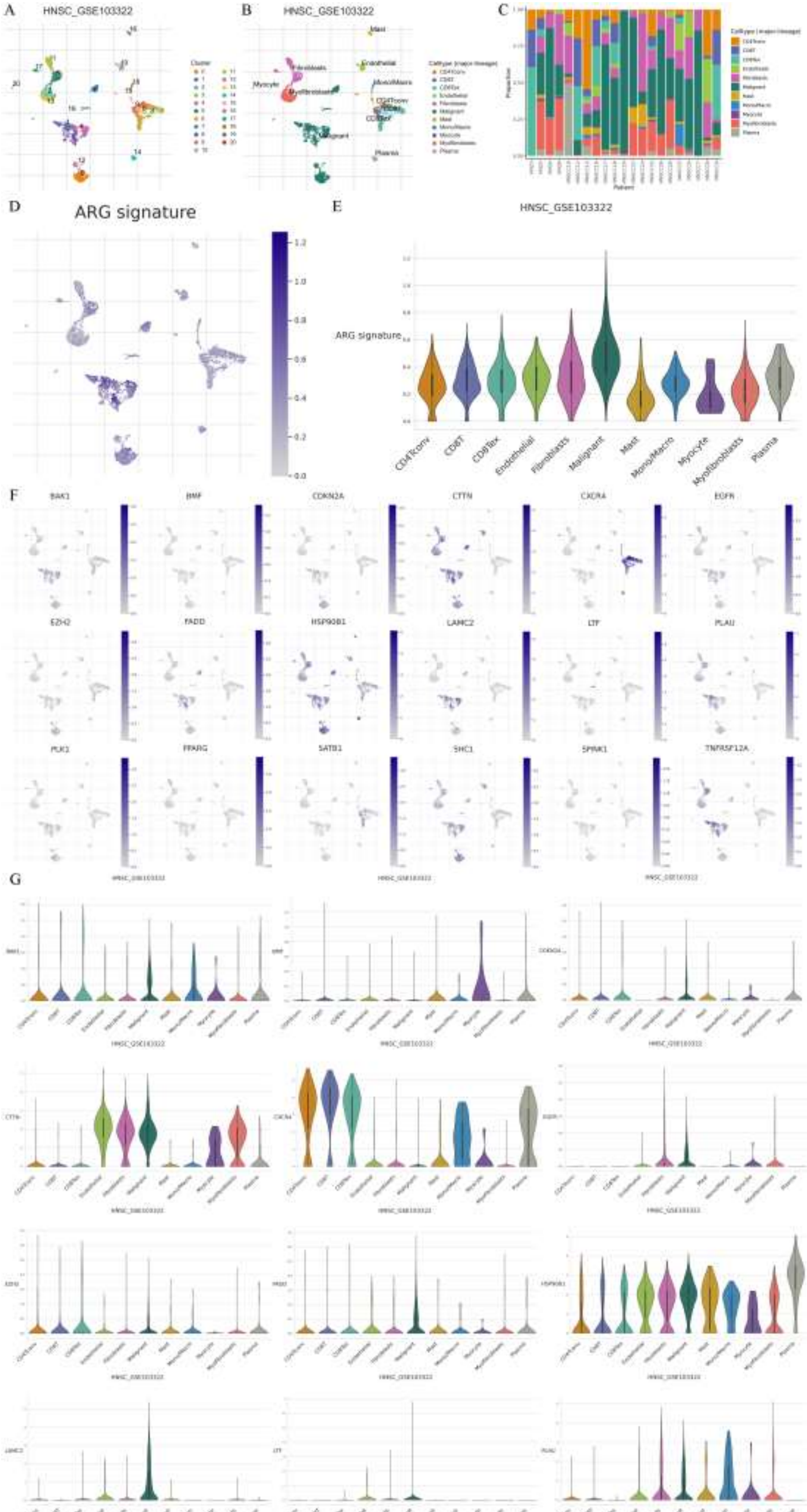
A
HNSC_GSE103322
B
HNSC_GSE103322
C
D
ARG signature
E
HNSC_GSE103322
ARG signature
CD4Tconv
CD8T
CD8Tex
Endothelial
Fibroblasts
Malignant
Mast
Mono/Macro
Myocyte
Myofibroblasts
Plasma
F
BAK1
BMF
CDKN2A
CTTN
CXCR4
EGFR
EZH2
FADD
HSP90B1
LAMC2
LTF
PLAU
PLK1
PPARG
SATB1
SHC1
SPINK1
TNFRSF12A
G
