# Supplementary material for: Identification of a new anoikis-related gene signature for prognostic significance in head and neck squamous carcinomas
Source: Medicine (Baltimore). 2023 Sep 8;102(36):e34790. doi: 10.1097/MD.0000000000034790 (PMC10489427; doi:10.1097/MD.0000000000034790)
Supplement: Supplementary file 2 [file medi-102-e34790-s002.pdf]

Figure S2: Comparison of the ARG signature with other signatures. Kaplan-Meier survival curves of Jiang signature (A), Ming signature (B), Yang signature (C), Zhang signature (D), and Zhao signature (E). Time-dependent ROC curves of Jiang signature (F), Ming signature (G), Yang signature (H), Zhang signature (I), and Zhao signature (J). (K) Restricted mean survival time curve for all signatures. (L) C-index for all signatures.

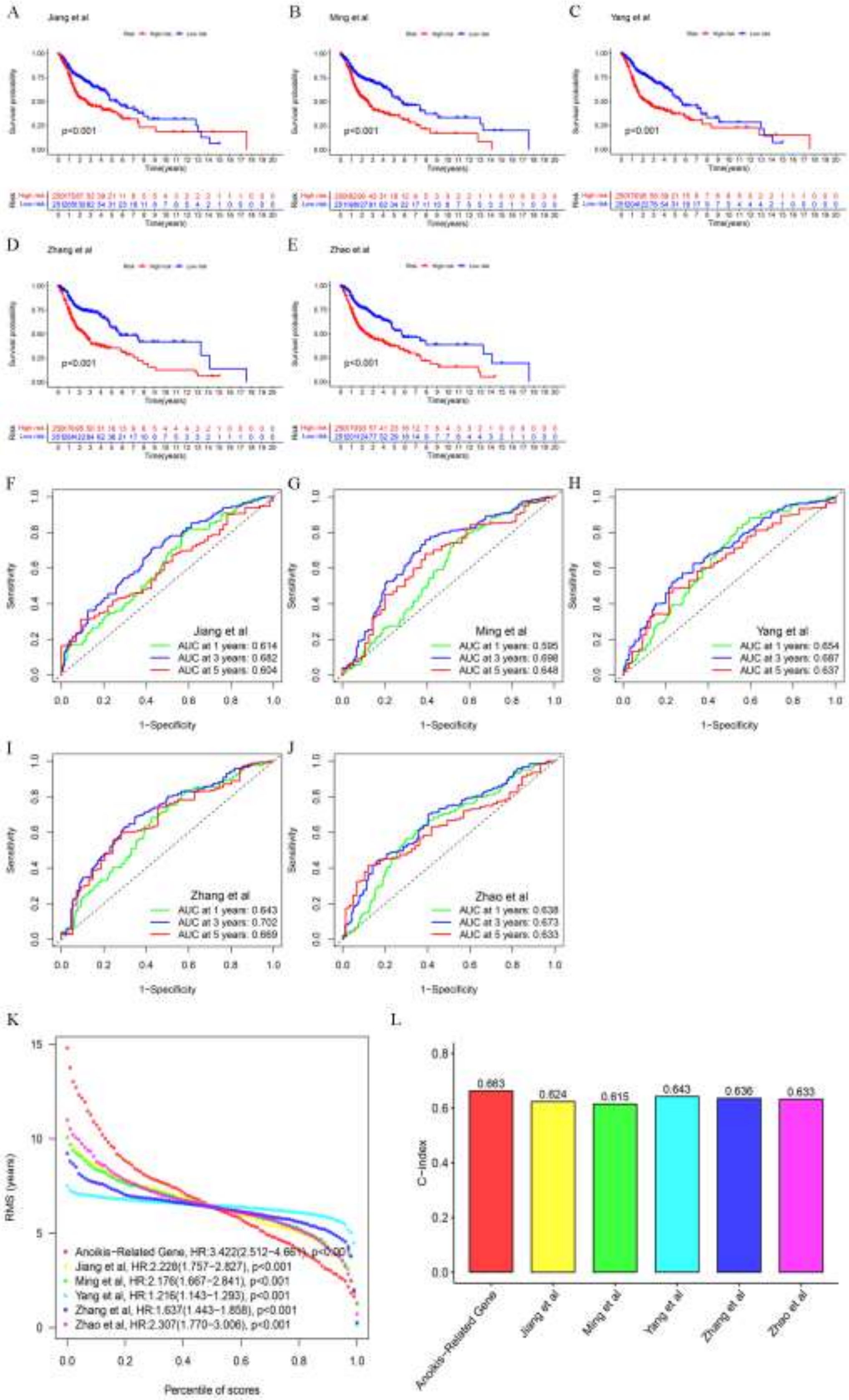
A
Jiang et al
p<0.001
B
Ming et al
p<0.001
C
Yang et al
p<0.001
D
Zhang et al
p<0.001
E
Zhao et al
p<0.001
F
Jiang et al
AUC at 1 years: 0.614
AUC at 3 years: 0.682
AUC at 5 years: 0.604
G
Ming et al
AUC at 1 years: 0.595
AUC at 3 years: 0.698
AUC at 5 years: 0.648
H
Yang et al
AUC at 1 years: 0.654
AUC at 3 years: 0.687
AUC at 5 years: 0.637
I
Zhang et al
AUC at 1 years: 0.643
AUC at 3 years: 0.702
AUC at 5 years: 0.669
J
Zhao et al
AUC at 1 years: 0.638
AUC at 3 years: 0.673
AUC at 5 years: 0.633
Sensitivity
1−Specificity
K
RMS (years)
Percentile of scores
Jiang et al, HR:2.228(1.757−2.827), p<0.001
Ming et al, HR:2.176(1.667−2.841), p<0.001
Yang et al, HR:1.216(1.143−1.293), p<0.001
Zhang et al, HR:1.637(1.443−1.858), p<0.001
Zhao et al, HR:2.307(1.770−3.006), p<0.001
L
C−index
0.663
0.624
0.615
0.643
0.636
0.633
Anoikis−Related Gene
Jiang et al
Ming et al
Yang et al
Zhang et al
Zhao et al
